# Context-based preprocessing of molecular docking data

**DOI:** 10.1186/1471-2164-14-S6-S6

**Published:** 2013-10-25

**Authors:** Ana T Winck, Karina S Machado, Osmar Norberto de Souza, Duncan D Ruiz

**Affiliations:** 1 GPIN - Grupo de Pesquisa em Inteligência de Negócio, PPGCC, Faculdade de Informática, PUCRS Av. Ipiranga, 6681 - Prédio 32, sala 628, 90619-900, Porto Alegre, RS, Brasil; 2 LABIO - Laboratório de Bioinformática, Modelagem e Simulação de Biossistemas, PPGCC, Faculdade de Informática, PUCRS Av. Ipiranga, 6681 - Prédio 32, sala 602, 90619-900, Porto Alegre, RS, Brasil

## Abstract

**Background:**

Data preprocessing is a major step in data mining. In data preprocessing, several known techniques can be applied, or new ones developed, to improve data quality such that the mining results become more accurate and intelligible. Bioinformatics is one area with a high demand for generation of comprehensive models from large datasets. In this article, we propose a context-based data preprocessing approach to mine data from molecular docking simulation results. The test cases used a fully-flexible receptor (FFR) model of *Mycobacterium tuberculosis *InhA enzyme (FFR_InhA) and four different ligands.

**Results:**

We generated an initial set of attributes as well as their respective instances. To improve this initial set, we applied two selection strategies. The first was based on our context-based approach while the second used the CFS (Correlation-based Feature Selection) machine learning algorithm. Additionally, we produced an extra dataset containing features selected by combining our context strategy and the CFS algorithm. To demonstrate the effectiveness of the proposed method, we evaluated its performance based on various predictive (RMSE, MAE, Correlation, and Nodes) and context (Precision, Recall and FScore) measures.

**Conclusions:**

Statistical analysis of the results shows that the proposed context-based data preprocessing approach significantly improves predictive and context measures and outperforms the CFS algorithm. Context-based data preprocessing improves mining results by producing superior interpretable models, which makes it well-suited for practical applications in molecular docking simulations using FFR models.

## Background

Data preprocessing is a major step in data mining. Although time-consuming, it improves data quality so they can be properly mined, thus producing more accurate, interpretable, and applicable models. Many techniques can be applied to data preprocessing [1], including data cleaning, data integration, and data transformation. In predictive machine learning problems, there is an input *x *and an output *y*; the task is to learn how to map the input to the output. Such a mapping can be defined as a function *y *= *g*(*x|θ*) where *g*(.) is the model and *θ *its parameters [2].

Although we can find numerous algorithms for prediction, many of them only work by producing a predictive function that indicates to which target value the objects belong. However, in some data mining problems, it is necessary to have a better comprehension of the induced models. Decision trees are models well understood by users. Indeed, Freitas et al. [3] support the use of decision trees models, instead of black box algorithms, to represent, graphically, patterns revealed by data mining, for example, Support Vector Machine (SVM) or Neural Networks models. Still according to these authors [3], the hierarchical structure developed can emphasize the importance of the attributes used for prediction.

The incorporation of context-aware data preprocessing to improve mining results is an active area of research. Baralis et al. [4] develop the CAS-Mine: a context-based framework to extract generalized association rules, providing a high-level abstraction of both, user habits and service characteristics, depending on the context. Nam et al. [5] discuss how the context can help classify the face image. Although these authors discuss the importance of considering the context in data mining applications while they develop their work according to a context-aware definition, the context involved is intrinsically specific to each working background. Hence, their methodologies are not suitable to the molecular docking simulations context explored in this work.

There are many areas of application where a comprehensible model is fundamental to its proper use. In bioinformatics, only a set of data and a set of data mining models may not be enough. The data and the results must represent the context in which they are embedded. Bioinformatics is a clear example of where we believe data preprocessing is instrumental. Our contribution is within the context of rational drug design (RDD). The interactions between biological macromolecules, called receptors, and small molecules, called ligands, constitute the fundamental principle of RDD. *In-silico *molecular docking simulations, an important phase of RDD, investigate the best bind pose and conformation of a ligand into a receptor. The best ligands are tested by *in-vitro *and/or *in-vivo *experiments. If the results are promising, a new drug candidate can be produced [6]

A proper data preprocessing may induce decision-trees models that are able to identify important features of the receptor-ligand interactions from molecular docking simulations. In the present work, we propose and apply a predictive regression decision-tree on the context-based preprocessed data from docking results and show that bioinformaticians can easily understand, explore, and apply the induced models. We apply four preprocessing techniques. Firstly, we produce and arrange all attributes based on the domain knowledge. Secondly, still based on a context domain, we improve the input by selecting two appropriate features. Thirdly, we apply a conventional machine learning feature selection to the initial set of attributes. Finally, we combine the feature selection generated using the first and second strategies with those from the third strategy. We assess the results for the model's accuracy and interpretability. Then, we demonstrate how a careful and value-added data preprocessing can produce more effective models.

## Methods

### The molecular docking context

Interaction between drug candidates (ligands) and target proteins (receptors), through molecular docking simulations, is the computational basis of RDD. Given a receptor, molecular docking simulations sample a large number of orientations and conformations of a ligand inside its biding site. The simulations also evaluate the Free Energy of Binding (FEB) and rank the orientations/conformations according to their FEB scores[7]. The majority of molecular docking algorithms only consider the ligand as flexible, whereas the receptor remains rigid, due to the computational cost involved in considering the receptor's explicit flexibility. However, biological macromolecules, like protein receptors, are intrinsically flexible in their cellular environment. The receptor may modify its shape upon ligand binding, moulding itself to be complementary to the ligand [8]. This increases favourable contacts and reduces adverse interactions, which, in turn, minimizes the total FEB [9]. Therefore, it is important to consider the receptor's explicit flexibility in molecular docking simulations.

In this work, we model the full receptor explicit flexibility in the molecular docking simulations [10] using a set of different conformations for the receptor, generated by molecular dynamics (MD) simulations [11]. This type of representation, named a fully-flexible receptor (FFR) model [10], results in the need of executing large numbers of docking simulations and voluminous results to be analysed. Actually, one of the current major challenges in bioinformatics is how to handle large amounts of data [12], or big data [13].

### Data modelling and acquisition

The InhA enzyme from *Mycobacterium tuberculosis *(Mtb) [14] is the target protein receptor in this work. It contains 268 amino acid residues and 4,008 atoms. The 3D structure (PDB ID: 1ENY) of the crystal, rigid receptor [14], was retrieved from the Protein Data Bank [15]. The FFR model of InhA (FFR_InhA) contains 3,100 snapshots from a 3.1 ns MD simulation [11]. Machado et al. [10] performed molecular docking simulations of FFR_InhA against each of the four different ligands: TCL [16], PIF [17], ETH [18] and NADH [14].

All docking results and snapshots of the FFR_InhA model were stored into a proper repository [19]. We developed this repository to integrate FFR models and docking results, allowing users to query the database from different points of view [20]. In fact, queries can traverse relationships between receptors and ligands' atoms and vice-versa, including their conformations and 3D coordinates. This repository enables us to produce effective inputs to use in different data mining tasks with their corresponding algorithms.

### Attributes arrangements

A major objective of this work is to reduce the number of snapshots used as input in docking simulations of a FFR model against a given ligand. In this sense, by mining the data from the FFR model and its docking results, we expect to select a subset of all available receptor conformations that are most relevant and capable of indicating whether a given ligand is a promising compound. Machado et al. [21][22] demonstrated how data mining can address this question. Winck et al. [23] obtained encouraging results by applying a context-based preprocessing to data mining of biological text. Hence, we focus our efforts on context-based data preprocessing. In our database [19] there are many available features. Choosing the most important ones impacts directly the choice of the proper data mining algorithm. Predictive data mining task is defined by the target attribute [24]. In the following sections we define the target and predictive attributes of the domain-specific knowledge of this work.

### Target attribute definition

**Figure 1:**
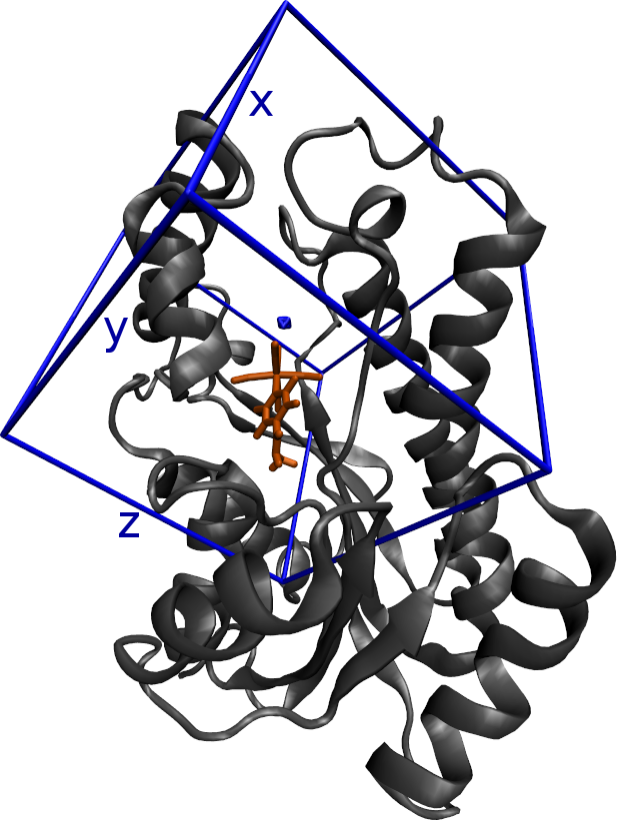
**3D-Grid considering the InhA receptor and the PIF ligand**. This 3D-Grid has 60.0 Å of size in axes *x, y *and *z*. The distance between each point is 0.375 Å.

One way to evaluate a molecular docking simulation with AutoDock3.0.5 [25] is by examining the values of the resulting free energy of binding (FEB): the most negative FEB values generally indicate the best receptor-ligand binding affinity. AutoDock3.0.5 predicts the bound conformations of a ligand to a receptor. It combines an algorithm of conformation search with a rapid grid-based method of energy evaluation [25]. The AutoGrid module of AutoDock3.0.5 pre-calculates a 3D energy-based grid of interactions for various atom types. Figure 1 shows an example of the grid box used in this work.

We adopt the FEB as our target attribute because it discriminates docking results. There is no consensus about what is the reasonable range of FEB values. Each ligand has to be considered and evaluated individually. Analysis of FEB values from the docking simulations of the FFR_InhA with the four ligands produced different ranges of minimum, maximum and average FEB values (Table 1).

**Table 1:** Range of FEB (Kcal/mol) values to each ligand considered.

Ligand	Min FEB	Max FEB	Avg FEB
NADH PIF TCL ETH	-20.61 -11.22 -10.01 -8.22	-0.02 -0.01 -0.73 -5.27	-9.23 *± *4.54 -9.09 *± *1.63 -8.17 *± *1.28 -6.37 *± *0.34

Analysis of Table 1 shows that the difference between the lowest and highest values is very subtle. Although we have an absolute difference between these extreme values (for instance, for ETH it is -2.95 kcal/mol), there are many instances where the decimal value varies sometimes a difference between two FEB values, for instance for ETH, 6.71 and 6.03 can be significant. In previous work, Machado et al. [26][27] using the same four ligands, discretized the FEB values using three different procedures: by equal frequency, by equal width and an original method based on the mode and standard deviation of FEB values. The authors split the FEB into five classes: Excellent, Good, Regular, Bad, and Very Bad. This preprocessing step generated the input data upon which the J48 decision tree algorithm was executed. The resulting performance's measures showed that discretization by equal frequency is not satisfactory. That by equal width had good evaluation for two of the four ligands only [27]. In these cases, J48 did not generate legible trees. Discretization by the mode and standard deviation, however, had better performance's measures for two ligands and produced more legible decision trees for all four ligands[27]. Although the J48 algorithm produced encouraging results, we found it challenging to discretize FEB values whose differences were particularly small. For instance, it was difficult to decide if a FEB value of -8.10 kcal/mol is a Good or Regular FEB since the difference to the next FEB value was -0.10 kcal/mol only. Because of the significance of the decimal values we may have an important loss of information when applying this discretization to FEB values. Therefore, the FEB value is taken as real values, which implies the use of a regression predictive task of data mining.

### Predictive attributes definition

According to Jeffrey [28] and da Silveira et al. [29] meaningful contact between two atoms can be established on a distance as large as 4.0 Å. In molecular docking, the FEB value is dependent on the shortest distance between atoms of the receptor's residues and ligands. This is because receptor-ligand atoms' pairs within 4.0 Å engage in favourable hydrogen bonds (HB) and hydrophobic contacts (HP) [28]. Hence, for each receptor (*R*) residue, we calculate the Euclidean distance (*ED*) between their atoms and the atoms of the ligand (*L*). We define *min*(*Dist_R,L_*) as the predictive attribute representing the shortest distance between the ligand and the receptor's residues. Thus, *min*(*Dist_R,L_*) with a 4.0 Å threshold indicates the presence of receptor-ligand favourable contacts (HBs and HPs). Only *min*(*Dist_R,L_*) is recovered from the repository [19]. If we used all receptor-ligand distances the input file would have an enormous amount of attributes, for example, for the PIF ligand which has 24 atoms, the entry would have more than 96,000 attributes! This number of predictive attributes would generate model trees with huge amounts of nodes, and, therefore, would not be interpretable. Each of the 3,100 snapshots of the FFR_InhA will have 268 attributes. We repeat the same procedure for all four ligands. In the end, we have one preprocessed input for each of the four ligands.

### Data preprocessing strategies

Our database does store the FFR_InhA which contains 3,100 snapshots (*Sn*), each with 4,008 atoms (*AtR*). It totalizes *Sn × AtR *= 12, 242, 800 receptor coordinates (*CoordR*). Because each docking simulation is made of 10 runs, we obtain 31,000 docking results for each ligand. However, some docking simulations runs did not converge or had positive FEB values. It occurs when the number of runs and the number of cycles defined as parameter to the algorithm are not enough to find a good position to bind the ligand into the receptor. The docking simulations were performed using the Simulated Annealing (SA) algorithm, which makes its conformation exploration using the Monte Carlo approach. Since in each step of execution a random movement is applied inside the binding site, sometimes the ligand keeps in a non-favourable position during the number of runs established. If it happens during many runs, the docking result does not converge, that is, it does not present any interaction position/energy in the end of the execution of a given experiment. We considered these data as outliers and did not include them in the preprocessing step. We also defined the parameter *ValDoc *as the total number of valid docking simulations per ligand. Since *AtLig *is the number of atoms of each ligand, the sum of the product *AtLig × ValDoc *for all four ligands determines the total number of ligand coordinates (*LigCoord*). In summary, we have:

*• CoordR *= 3, 100 *× *4, 008 = 12, 424, 800 records

*• LigCoord_NADH _*= 52 *× *11, 284 = 586, 768 records

*• LigCoord_TCL _*= 18 *× *28, 370 = 510, 660 records

*• LigCoord_PIF _*= 24 *× *30, 420 = 730, 080 records

*• LigCoord_ETH _*= 13 *× *30, 430 = 395, 590 records

*• LigCoord *= 586, 768 + 510, 660 + 730, 080 + 395, 590 = 2, 223, 098 records

### Data generation

To generate an initial dataset we need to combine the 12,424,098 *CoordR *and the 2,223,098 *LigCoord*, calculate their interactions, and find their respective *min*(*Dist_R,L_*). For that, we developed the Dataset algorithm. It executes the first preprocessing step by handling the input data and by producing the best receptor-ligand interactions stored in an output file: the [*Input*] matrix. [*Input*] contains *ValDoc *lines and 269 columns. The first 268 columns contain the 268 receptor residues *min*(*Dist_R,L_*). To generate a proper dataset for data mining, we aggregated a target attribute in the last column, which is the corresponding FEB value. It is important to emphasize that, at this stage, *min*(*Dist_R,L_*) can have any positive value.

- Dataset Algorithm -

Let*R *be a receptor

Let*L *be a ligand

Let*t *be a snapshot of*R*

Let*r *be a residue of*R*

Let*a *be an atom in*t *snapshot

Let*l *be an atom in*L*

Let*Dist *be the distance between*L *and*R *atoms in*t*

Let*DistanceMatrix *be a matrix where each line corresponds to a residue*r *and each cell corresponds to the distance between*a *and*l*

Let*Result *be a matrix that stores for each*t *snapshot, all minimum distances between*a *and*l*

Let*Input *be a matrix containing*Result *and, for each*t*, its respective FEB value

FOR each*t *in*Total_Snapshots_R_*

    [*Result*]_* _←*null*

    FOR each*r *in*Total_Residues_R_*

        [DistanceMatrix_*,*_]*← null*

        FOR each*a *in*Total_Atoms_Residue_Snapshot_R,t_*

            FOR each*l *in*Total_Atoms_Ligand_L_*

                $Dist_{Ri,Li}\leftarrow \sqrt{{\left(x_{R}-x_{L}\right)}^{2}+{\left(y_{R}-y_{L}\right)}^{2}+{\left(z_{R}-z_{L}\right)}^{2}}$

                *Dist_Ra,Ll _*←*ED*(*R, L*)

                [*DistanceMatrix_a,l_*]*← Dist_Ra,Ll_*

            ENDFOR

        ENDFOR

        [*Result_t,r_*]*← min*([DistanceMatrix_r,*_])

    ENDFOR

    [Input_t,*_]*← *[Result_t,* _+*FEB_L_*]

ENDFOR

### Dataset improvement

The initial dataset generated by the Dataset Algorithm contains 268 predictive attributes and a target attribute. To help improve the models induced by the data mining task, we must reduce further the amount of features. Jeffrey [28] states that the largest distance value that allows a meaningful contact between receptor and ligand atoms is 4.0 Å. The feature selection strategy in Dataset Algorithm includes distances higher than 4.0 Å. This means that the corresponding receptor residue does not establish a favourable contact with any of the ligand atoms [29]. If there is not a contact in any docking results, it is improbable that this attribute can adequately predict the FEB value. Therefore, we removed all attributes (residues) with shortest distances above the 4.0 Å threshold. Context-FS Algorithm generates a new input from the [*Input*] matrix output produced by Dataset Algorithm. To compare our context-based feature selection with a well-known machine learning feature selection algorithm, we generated one more dataset seeking to improve the initial one produced by the Dataset Algorithm. We believe that a subset of representative attributes can improve further the mining results.

- Context-FS Algorithm -

Let*R *be a receptor

Let*t *be a snapshot of*R*

Let*r *be a residue of*R*

Let*Input *be a produced by the Dataset Algorithm

Let*InputFS *be a result after our context-based feature selection

FOR each*r *in*Total_Residues_R_*

    IF*min*([Input_*,r_])*≤ *4

        FOR each*t *in*Input*

            [*InputFS_t,r_*]*← *[*Input_t,r_*]

        ENDFOR

    ENDIF

ENDFOR

FOR each*t *in*InputFS*

        [InputFS_t,*_]*← *[*Input_t,r+1_*]

ENDFOR

Only a limited number of the existing feature selection algorithms can work effectively on regression predictive tasks. Among these, the Correlation-based Feature Selection (CFS) [30] algorithm implemented in Weka [24] can perform feature selection on our datasets. Therefore, we applied CFS to each input generated by Dataset Algorithm, with a different input for each of the four ligands. CFS is based on a filter approach that ranks features according to a correlation-based heuristic evaluation function 1. It looks for a subset that contains features uncorrelated with each other, but highly correlated with the target attribute.

(1) $$M_{s}=\frac{kbarr_{cf}}{\sqrt{k+k\left(k-1\right)r_{ff}^{-}}}$$

where: *M_S _*is a heuristic of a subset *S *that contains *k *features; *barr_cf _*is the mean feature-target correlation (*f *∈ *S*) and $r_{ff}^{-}$ is the average feature-feature inter-correlation. Equation 1 forms the core of CSF [30]. Table 2 shows the number of attributes selected after applying our feature selection methodology to the original dataset. Additionally, we generated one more dataset (Table 2, fourth column) which combines the features selected by the Context-FS Algorithm with those selected by CFS [30].

**Table 2:** Number of attributes selected after applying feature selection approaches.

Ligand	Context-FS Algorithm	CFS	Context-FS Algorithm ∪ CFS
NADH	84	17	93
TCL	106	14	114
PIF	104	16	108
ETH	105	6	111

### Mining and evaluation the preprocessed data

Regression is a data mining task suitable to problems for which the attribute to be predicted is continuous. Since our target attribute is numeric, regression is the technique applied to the mining experiments in this study. Our models must be understandable and must also represent well the context in which they are inserted. Decision trees are algorithms that cover these needs and also can be applied to both classification and regression problems. The results are regression or classification models arranged in a tree structure. Decision trees can be applied to predict both continuous and discrete values. For continuous values, there are two main types of trees: regression trees and model trees. In regression trees, each leaf node stores a continuous-valued prediction, which is the average of the target attribute for the training tuples. In model trees, each leaf stores a regression model called Linear Model (LM), which is a multivariate linear equation for the target attribute [1]. Our goal is to induce models that indicate residues distances to predict a given FEB value. We expect our model to help us discover whether a snapshot, when docked to a given ligand, will lead to favourable estimated FEB values. For this, we use the M5P [31] machine learning model tree algorithm.

### Evaluation of the induced models

There are several measures to verify if the induced models generated are acceptable numerical predictions. They are called predictive measures. In the case of model tree algorithms, the most widespread measures are: root mean-squared error (RMSE, equation 2), mean absolute error (MAE, equation 3), and correlation coefficient [24]. Smaller values of RMSE and MAE are indicators of better models. All of these measures make use of the predicted values *p*_1 _. . . *p_n _*and the actual values *a*_1 _. . . *a_n_*.

(2) $$RMSE=\sqrt{\frac{{\left(p_{1}-p_{a}\right)}^{2}+\ldots +{\left(p_{n}-a_{n}\right)}^{2}}{n}}$$

(3) $$MAE=\frac{\left|\left(p_{1}-p_{a}\right)+\ldots +|\left(p_{n}-a_{n}\right)\right|}{n}$$

The correlation coefficient (Equation 4) measures the statistical correlation between *a *and *p*. The values range from 1, for perfectly correlated results, to 0, when there is no correlation, and to -1, for an inverse perfect correlation. We look for perfectly correlated results or correlation coefficients closer to 1.

(4) $$Correlation=\frac{S_{PA}}{\sqrt{S_{P}S_{A}}}$$

Where $S_{PA}=\frac{\sum_{i}\left(p_{i}-\bar{p}\right)\left(a_{i}-\bar{a}\right)}{n-1},S_{p}=\frac{\sum_{i}{\left(p_{i}-\bar{p}\right)}^{2}}{n-1}$, and $S_{a}=\frac{\sum_{i}{\left(a_{i}-\bar{a}\right)}^{2}}{n-1}$, being that *ā *and $\bar{p}$ are the corresponding *a *and *p *averages.

In addition to these measures, some investigations also make use of the model interpretability metric, which is the number of nodes in the model tree. The model tree with the smallest amount of nodes generates the best interpretable models [32].

### Evaluation based on the context

**Figure 2:**
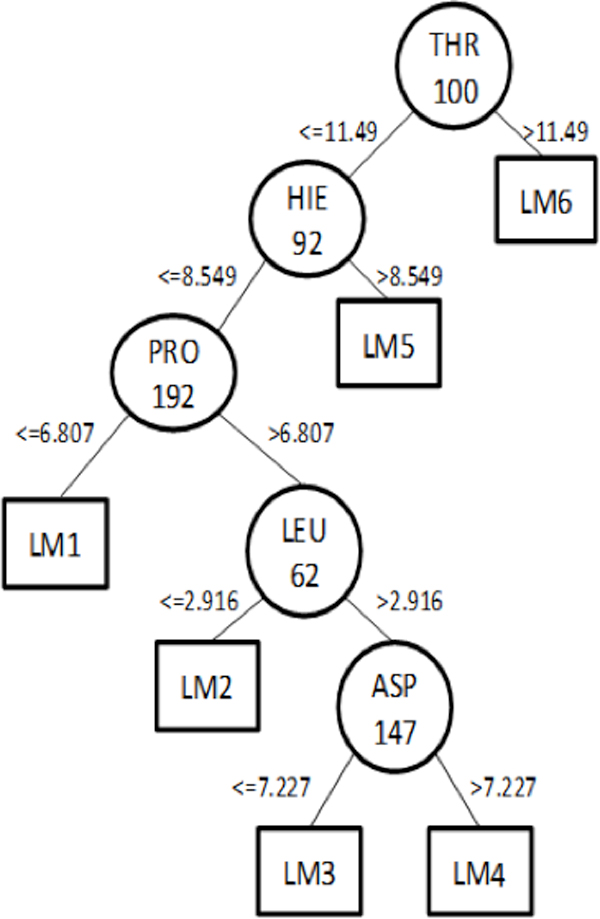
**Model Tree generated by the M5P Algorithm**.

The measures shown in the previous section were used during the evaluation of the models generated. However, as we are interested in the usefulness of the induced models, we propose a new context-based measure. We also analyze the induced model trees and their contents. Figure 2 shows a model tree generated upon application of our context-based preprocessing (Context-FS Algorithm) to NADH. This model contains five non-leaf nodes, each representing a selected amino acid residue, and six LMs. Equation 5 depicts the sixth LM (LM6) composed of a selected number of predictive attributes (receptor residues) weighted by their effect in the target attribute (FEB) plus a constant value.

(5) $$FEB=\;\begin{array}{c} -0,0009\times SER12+0,9405\times PHE22\\ +0,0013\times THR38+0,0035\times ASP63\\ +0,0006\times HIE92+0,002\times THR100\\ -0,5005\times GLY101-0,0004\times ALA123\\ -0,0015\times ASP147+0,0024\times THR161\\ +0,0017\times LEU167+1,094\times GLY191\\ +0,0037\times PRO192+0,0015\times ILE193\\ +0,0003\times ILE201\\ -20,6455 \end{array}$$

We evaluate the models taking into account the receptor residues present in both the non-leaf nodes and the LMs, bearing in mind that the docking software calculates the FEB value only for the residues within the grid box around the receptor binding site (Figure 1). Consequently, if we are inducing model trees to predict FEB values, models that consider residues located outside the grid box have no direct significance. Usually, a specialist defines which residues belong to the receptor active site. These residues shape the active site for the complementary ligand binding. For InhA, the specialist selected 52 residues, here denoted by *ESR*. Subsequently, by inspecting each model, we identified which model's residues (*MR*) appear in the tree or the LMs (Figure 2 and Equation 5). Now we are able to evaluate *MR *and compare it with *ESR *by calculating the Precision (Equation 6), Recall (Equation 7) and F-score (Equation 8) measures [1].

(6) $$Precision=\frac{\left|\left\{Relevant\right\}|\cap |\left\{Retrieved\right\}\right|}{\left|\left\{Retrieved\right\}\right|}$$

(7) $$Recall=\frac{\left|\left\{Relevantes\right\}|\cap |\left\{Retrieved\right\}\right|}{\left|\left\{Relevant\right\}\right|}$$

(8) $$F-score=\frac{recall\times precision}{\left(recall+precision\right)/2}$$

In the context of this analysis:

• |{*Relevant*}*| ∩ |*{*Retrieved*}| can be defined as *ESR ∩ MR*

• *|*{*Relevant*}*| *can be defined as *ESR*

• {*Retrieved*}*| *can be defined as *MR*

## Results

We evaluated the models by means of the predictive and context measures presented. The measures were applied separately to each of the four distinct ligands; NADH, PIF, TCL, and ETH. For each one of them, we observed the four data preprocessing strategies:

1. The results obtained by the initial dataset, generated by Dataset Algorithm;

2. The results obtained by the context-based feature selection, generated by Context-FS Algorithm;

3. The results obtained by the feature selection generated by CFS [30];

4. The results obtained by combining both feature selection generated by Context-FS Algorithm and CFS (Table 2 fourth column).

The initial dataset was the first and, possibly, the most important context-based data preprocessing. Without the previous knowledge about the context, it would not be possible to generate an input that produces interpretable models as we expected. Based on the fact that the initial dataset was constructed considering minimum distances (*min*(*Dist_R,L_*)), our hypothesis is that the context-based data preprocessing we proposed, including feature selection, produces better results than using a worthy feature selection approach, where the context is not observed. Hence, we expected that the results from the third strategy would not be better than the others. On the other hand, we expected the context-based feature selection (second strategy) to give better results than the others. The second strategy was applied considering both, the context already employed in the initial dataset and context to select appropriate features. To evaluate the results in terms of their statistical significance, we applied the Friedman Test [33] with a significance level of *α *= 0.05. For the context measures (Table 3), we evaluated whether strategy 2 was significantly better than the others. In this case, we could assert that it was true since *p *= 0.014. We infer that our feature selection approach improves the initial results. Therefore, as we are interested in the quality of the induced models, our context-based measure can be considered as the most appropriate.

**Table 3:** Model evaluation predictive measures.

Ligand	Preprocessing Strategy	Evaluation			
		
		Nodes	Correlation	MAE	RMSE
NADH	1	15	0.9536	1.0030	1.3660
	2	5	0.9512	1.0189	1.4000
	3	6	0.9483	1.0578	1.4396
	4	9	0.9513	1.0211	1.3992

PIF	1	22	0.9685	0.3077	0.4071
	2	19	0.9692	0.3053	0.4022
	3	22	0.9653	0.3237	0.4264
	4	19	0.9686	0.3067	0.4060

TCL	1	12	0.9700	0.2396	0.3108
	2	19	0.9708	0.2364	0.3068
	3	15	0.9667	0.2508	0.3273
	4	24	0.9708	0.2369	0.3069

ETH	1	18	0.6086	0.2106	0.2665
	2	15	0.5999	0.2123	0.2687
	3	16	0.5566	0.2212	0.2790
	4	17	0.6047	0.2118	0.2675

In Table 3 we evaluate whether strategy 3 is significantly worse than the others. We got *p *= 0.040 for MAE and *p *= 0.054 for RMSE as significance levels, indicating that probably strategy 3 is really worse than strategies 1, 2 and 4. However many effort is needed to assess it. With respect of context measures (Table 4), we evaluate whether strategy 2 is significantly better than the other ones. In this case, we can assert it is true because we got *p *= 0.014. We infer that our feature selection approach improves the initial results. In doing so, once we are interested in the quality of the induced models, our context-based measure can be considered as the most appropriate.

**Table 4:** Model evaluation context measures.

Ligand	Preprocessing Strategy	Evaluation		
		
		Precision	Recall	F-score
NADH	1	0.1176	0.0385	0.0580
	2	0.4375	0.1346	0.2059
	3	0.3636	0.0769	0.1270
	4	0.1875	0.0576	0.0882

PIF	1	0.2143	0.1731	0.1915
	2	0.5294	0.3462	0.4186
	3	0.4667	0.1346	0.2090
	4	0.4571	0.3076	0.3678

TCL	1	0.1282	0.0962	0.1099
	2	0.4412	0.2885	0.3488
	3	0.4286	0.1154	0.1818
	4	0.3928	0.2115	0.2750

ETH	1	0.3939	0.2500	0.3059
	2	0.4375	0.2692	0.3333
	3	0.1250	0.0192	0.0333
	4	0.4516	0.2692	0.3373

It is noticeable in Tables 3 and 4 that the results are different for each ligand, despite employing the same strategy in the preprocessing. This is so because different ligands have different sizes, as well as different molecular interaction properties. They bind in different regions of the receptor's binding site. As a result, the target attribute FEB has different ranges of values for the distinct ligands (Table 1) and that is why the models are induced for individual ligands. Although they are not interchangeable, we expect them to be used to select ligands that belong to a similar class (high molecular similarity).

## Conclusions

Data preprocessing is a significant step in data mining. In data preprocessing, different techniques are applied to improve data quality such that the mining results are more accurate and better interpretable. There are many techniques available to preprocess data, mainly for model quality measures. However, some applications, like bioinformatics, often demand well-suited models. Hence, when the data mining process is based on the context involved, a context-based preprocessing can improve the quality of the induced models.

In this article, we presented a case of mining data from flexible receptor molecular docking simulations results. Here the goal was to identify features that could characterize the best fit of ligands into a given receptor. Our experiments were conducted considering the InhA receptor from the *M. Tuberculosis *and four distinct ligands: NADH, PIF, TCL, and ETH. We showed that an appropriate context-based data preprocessing could provide improved results.

We concentrated on four main preprocessing steps which: 1) consider the context to choose an initial set of attributes and the proper instances for each ligand input file; 2) perform feature selection on the initial dataset, taking into account the characteristics of the docking results from each ligand; 3) perform feature selection, for each ligand, based on the CFS machine learning algorithm; and 4) combine features selected by our context-based approach (Context-FS Algorithm) and those selected by the CFS algorithm. We hypothesized that mining the preprocessed data would provide better results, with respect to the original dataset, by using the second strategy.

We performed mining experiments using the M5P model tree algorithm implemented in Weka. The values of the RMSE error measure, as well as a context-based metric that considers the tree interpretability, suggested that we can obtain better results when using our feature selection approach (second strategy). Statistical analysis of the results, with the Friedman test, showed that our context-based approach significantly improves predictive measures while CFS worsens context measures. We concluded that data preprocessing, which considers the context involved, can improve the mining results and produce better interpretable models. As future studies, we plan to use the induced models, generated using the second strategy, to select the most promising subset of snapshots, out of a very large ensemble, for a given ligand.

## Competing interests

The authors declare that they have no competing interests.

## Authors' contributions

ATW and KSM executed the preprocessing for the data mining experiments, performed all the data mining experiments, evaluated the models results and wrote the first draft of the article. DDAR helped to conceive the test cases and to evaluate the models. ONS helped to analyze the results and to write the final version of the article. All authors read and approved the final manuscript.

## Author's information

ATW current address:

LaCA - Labortório de Computação Aplicada, Departamento de Computação Aplicada, Universidade Federal de Santa Maria (UFSM), Santa Maria, RS, Brasil.

KSM current address:

ComBi-L, Grupo de Biologia Computacional, Centro de Ciências Computacionais, Universidade Federal do Rio Grande (FURG), Rio Grande, RS, Brasil.

## Declarations

Publication of this article has been funded by the authors.

This article has been published as part of *BMC Genomics *Volume 14 Supplement 6, 2013: Proceedings of the International Conference of the Brazilian Association for Bioinformatics and Computational Biology (X-meeting 2012). The full contents of the supplement are available online at http://www.biomedcentral.com/bmcgenomics/supplements/14/S6.
